# Electrocardiogram Abnormalities Following Diphenhydramine Ingestion: A Case Report

**DOI:** 10.21980/J85H1P

**Published:** 2023-01-31

**Authors:** Patrick Bruss, Christine Bowman, Teagan Carroll

**Affiliations:** * ProMedica Monroe Regional Hospital, Department of Emergency Medicine, Monroe, MI

## Abstract

In the United States, tricyclic antidepressants (TCA) are commonly prescribed to treat psychiatric illnesses and neuropathic pain. This class of antidepressants has been found to cause pathognomonic electrocardiogram (ECG) changes in cases of overdose.^1^ Specifically, TCA’s cause a dominant terminal R wave in aVR and widening of the QRS complex due to their sodium channel blocking effect. Diphenhydramine, better known as Benadryl, is known to disrupt sodium channels in the same manner. In this case report, a 13-year-old female was brought into the emergency department (ED) after attempted suicide by diphenhydramine overdose. The patient presented with palpitations, nausea and confusion. She was agitated, tachycardic and exhibited opsoclonus. An ECG was performed upon the patient’s arrival which showed large terminal R waves in aVR along with large S waves in lead I indicating right axis deviation. Given the patient’s age and reported ingestion, it was highly suspicious that her symptoms and ECG changes were the result of a sodium channel blockade. Sodium bicarbonate was given, which resulted in notable ECG changes as well as symptomatic improvement. TCA’s, and similarly diphenhydramine, have sodium channel blocking properties which can be revealed by performing an ECG. Administration of sodium bicarbonate in the ED has been shown to be a successful treatment by reversing this sodium channel blockade.

**Topics:**

Tricyclic antidepressants, diphenhydramine, overdose, sodium channel blockage, sodium bicarbonate administration.

**Figure f1-jetem-8-1-v11:**
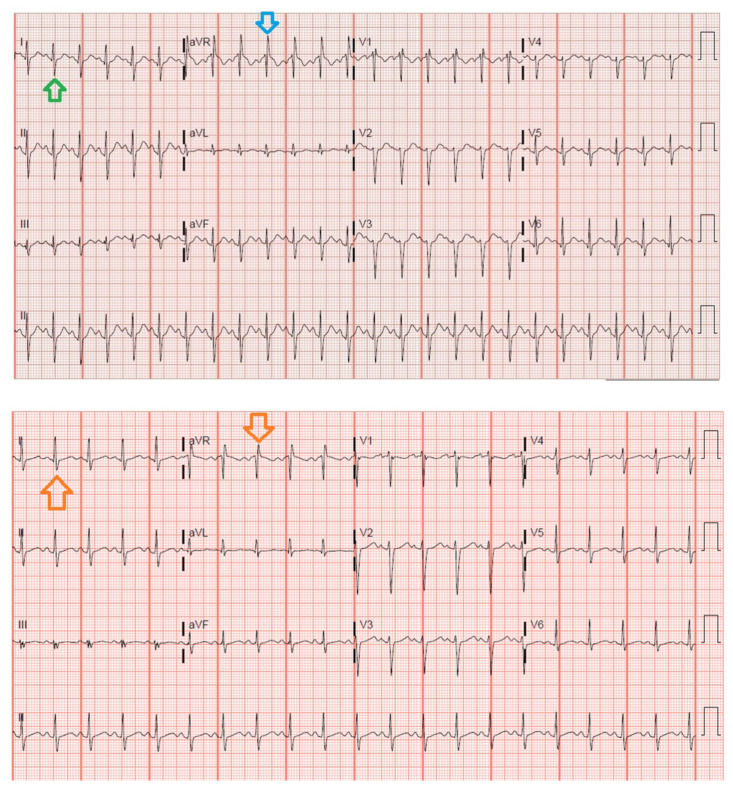


## Brief introduction

[Fig f1-jetem-8-1-v11]A 13-year-old female presented to the emergency department following a reported suicide attempt by way of diphenhydramine overdose. The patient complained of palpitations, nausea and confusion. She was agitated, tachycardic and exhibited opsoclonus. Her ECG was significant for terminal R waves in aVR and right axis deviation indicating a sodium channel blockade. Administration of sodium bicarbonate resulted in improvement of both the ECG abnormalities and patient’s symptoms. Sodium bicarbonate has been shown to be successful in reversing this sodium channel blockage in the ED.

## Presenting concerns and clinical findings

A 13-year-old female presents to the emergency department after reported suicide attempt by way of diphenhydramine overdose. The patient presented with palpitations, nausea and confusion. She was also agitated, tachycardic and exhibited opsoclonus.

## Significant findings

The blue arrow points to one of the terminal R waves in aVR, and the green arrow points to one of the large S waves in lead I, indicating right axis deviation. These findings are pathognomonic for sodium channel blockade. Due to the specific ECG findings and knowledge of diphenhydramine overdose, it was evident that these ECG findings were due to a cardiac sodium channel blockade. Sodium channels are essential within myocardial tissue to ensure the rapid upstroke of cardiac action potential, as well as rapid impulse conduction throughout cardiac tissue. Therefore, sodium channel blockers tend to exhibit significant dysrhythmic properties due to severe conduction disturbances.^2^ The blockage of the cardiac sodium channels appears as terminal R waves in aVR as well as terminal S waves in lead I due to delaying, and possibly blocking, the electrical conduction pathway of the heart. The orange arrows show resolution of terminal R wave in aVR and terminal S wave in lead I, after administration of sodium bicarbonate.

## Patient course

Given the patient’s age and presentation, reported ingestion of Benadryl, and ECG, providers suspected a sodium channel blockade. Terminal R waves in aVR along with terminal S waves in lead I, were the pathognomonic ECG changes noted. Sodium bicarbonate was promptly given and was effective in the reversal of the sodium channel blockage caused by the ingestion of diphenhydramine. After administration of sodium bicarbonate, there were obvious changes in the patient’s ECG, along with symptom improvement. Resolution of these ECG changes confirmed that the original ECG findings were caused by dysfunction of the electrical conduction pathway of the heart, due to sodium channel disturbance. In this case, there were not any complications following administration of sodium bicarbonate. The patient was admitted to the pediatric ICU overnight for observation, was medically cleared 24 hours later, and was evaluated by psychiatry.

## Discussion

This case report shows the efficacy of sodium bicarbonate administration, in light of sodium channel disruption secondary to diphenhydramine ingestion. Strengths of this case include prompt effective treatment and apparent resolution of original ECG findings and improvement of symptoms. The patient’s ECG on arrival showed terminal R waves in aVR and right axis deviation. TCAs are understood to cause these changes as well as prolongation of both the QRS and QTC by way of blocking the sodium channels within the heart. A prolonged QRS is defined as one greater than 100 ms, whereas a prolonged QTC is defined as one greater than 440 ms in men or 460 ms in women. Sodium bicarbonate causes an increase in pH as well as an increase in extracellular sodium which reverses this blockade.^3^ Another instance of a suspected diphenhydramine overdose successfully treated with sodium bicarbonate is that of a 13-month-old infant patient brought to the emergency department for tonic-clonic seizure activity following ingestion of diphenhydramine tablets. The infant’s ECG revealed a terminal R wave in aVR as well as wide-complex tachycardia, both of which were resolved following the administration of sodium bicarbonate.^4^ Blockade of the cardiac sodium channels appears as terminal R waves in aVR, defined as an R wave greater than 3 mm, and terminal S waves with an R/S ratio greater than 0.7. These findings are due to delay and possible blocking of the conduction pathway of the heart. The heart’s right side is more susceptible to sodium channel blockage, causing these characteristic ECG changes. Administration of sodium bicarbonate in the ED as well as the prehospital setting has been shown to successfully reverse this blockade.

## Supplementary Information








