# Supplementary figures and images for: Electrocardiogram Abnormalities Following Diphenhydramine Ingestion: A Case Report
Source: J Educ Teach Emerg Med. 2023 Jan 31;8(1):V11–3. doi: 10.21980/J85H1P (PMC10332767; doi:10.21980/J85H1P)

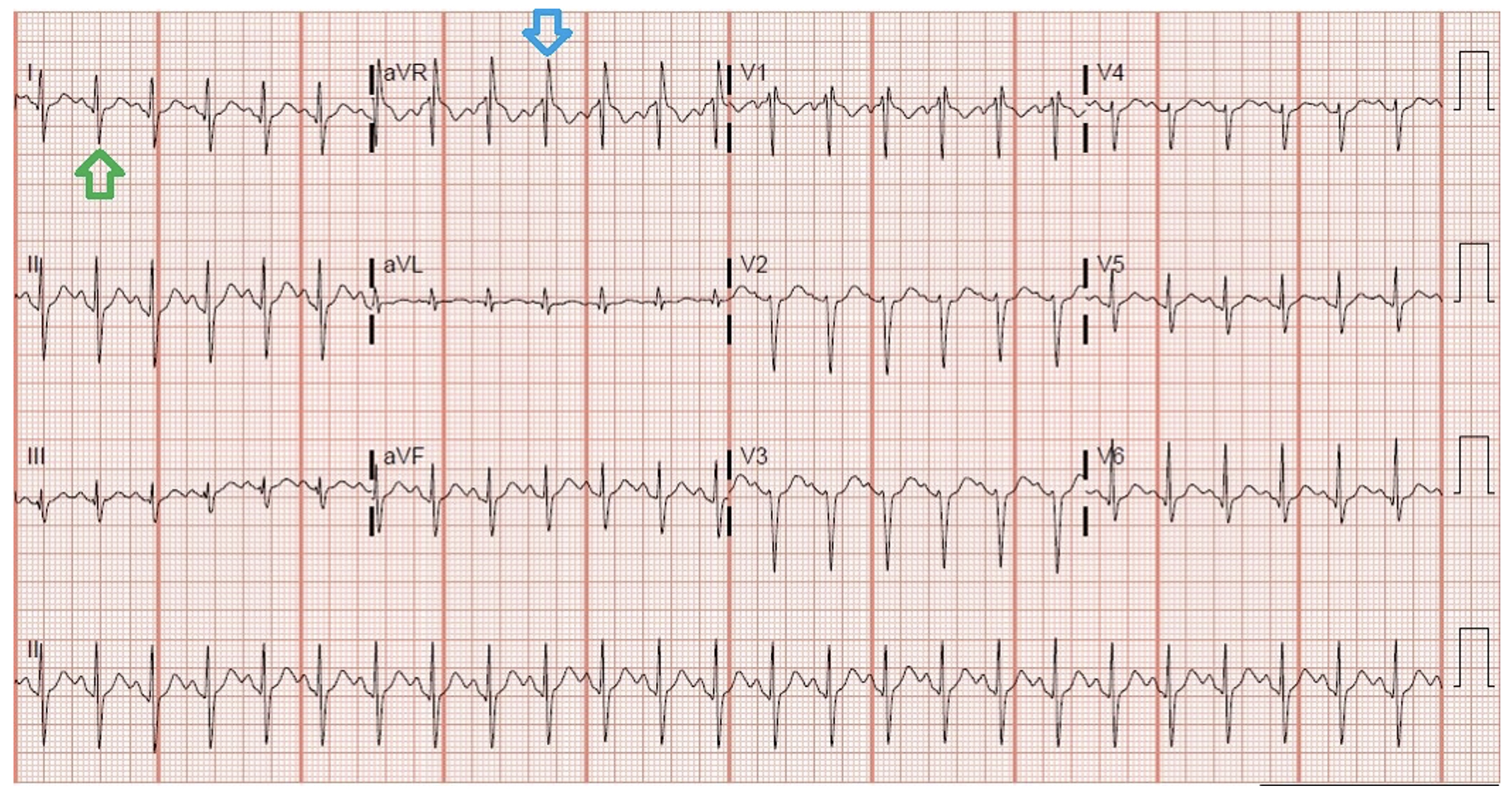

Supplement: Supplementary file 1 [file jetem-8-1-v11-supp1.jpg]

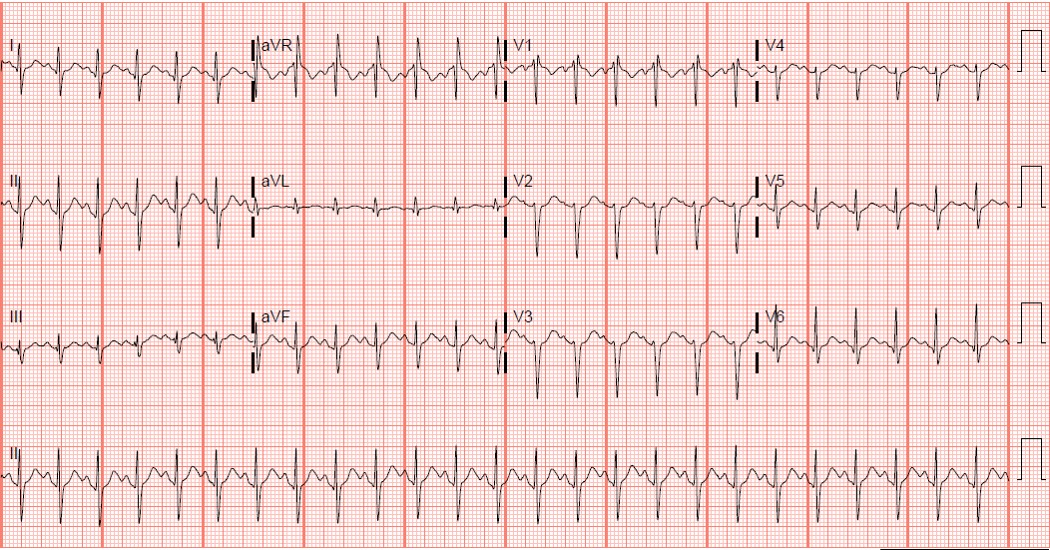

Supplement: Supplementary file 2 [file jetem-8-1-v11-supp2.jpg]

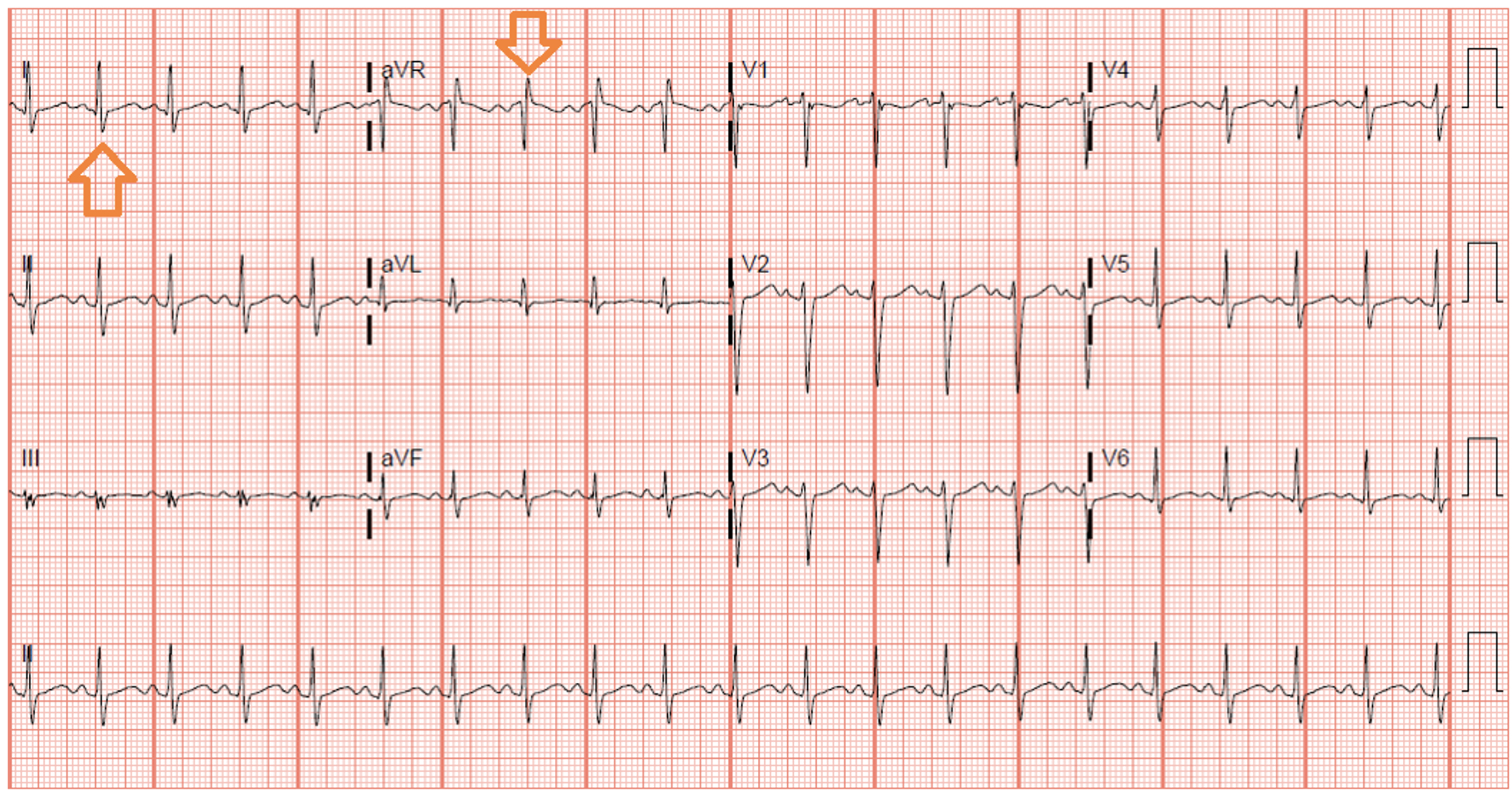

Supplement: Supplementary file 3 [file jetem-8-1-v11-supp3.jpg]

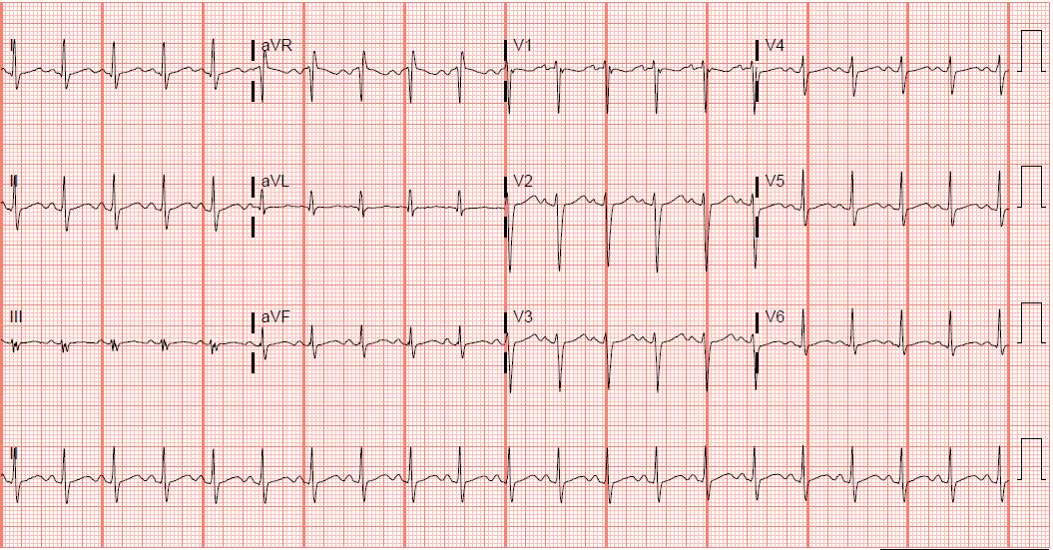

Supplement: Supplementary file 4 [file jetem-8-1-v11-supp4.jpg]
